# The Effects of the COVID-19 Pandemic on Cancer Staging in Patients Diagnosed With Head and Neck Cancer

**DOI:** 10.7759/cureus.34190

**Published:** 2023-01-25

**Authors:** Delanie P Mack, Horace Spencer, Kaidi Wang, Gary D Lewis

**Affiliations:** 1 Medicine: Radiation Oncology, University of Arkansas for Medical Sciences, Little Rock, USA; 2 Biostatistics, University of Arkansas for Medical Sciences, Little Rock, USA; 3 Radiation Oncology, University of Arkansas for Medical Sciences, Little Rock, USA

**Keywords:** prognosis, treatment, staging, cancer, head & neck, covid-19 retro

## Abstract

Purpose

The healthcare system across the world was forced to implement new policies, guidelines, and procedures due to the coronavirus disease 2019 (COVID-19) pandemic, which led many patients to make an impossible choice about their health. For various reasons, many patients chose to remain at home and delay any interaction at medical facilities to protect themselves or others from the virus. Patients managing chronic diseases faced unprecedented challenges during this period, and the long-term effects on these patient populations remain unclear. Oncology patients, specifically those diagnosed with head and neck cancers, require prompt diagnosis and initiation of treatment for better outcomes. While the overall impact of how the pandemic has affected oncology patients is unknown, this retrospective study examined how the staging of head and neck tumors at our institution has been impacted since the beginning of the pandemic.

Methods

Available patient data (from August 1, 2019, through June 28, 2021) were collected from medical records and compared to determine statistical significance. Patients were categorized into a Pre-pandemic group, Pandemic group, and Vaccine-approved group, and patient and treatment characteristics were analyzed to look for patterns. The pre-pandemic period was defined as the period from August 1, 2019, to March 16, 2020, the pandemic period was defined as the period from March 17, 2020, to December 31, 2020, and the vaccine-approved period was defined as the period from January 1, 2021, to June 28, 2021.

Results

Fisher’s exact tests were used to compare tumor, node, metastasis (TNM) staging distributions between the three groups. In the Pre-pandemic group, out of 67 patients, 33 patients (55.0%) were diagnosed with a T stage of 0-2 and 27 patients (45.0%) were diagnosed with a T stage of 3-4. In the Pandemic and Vaccine-approved groups, out of 139 patients, 50 patients (39.1%) were diagnosed with a T stage of 0-2 and 78 patients (60.9%) were diagnosed with a T stage of 3-4; these differences were statistically significant (P-value = 0.0426). The Pre-pandemic group had 25 patients (41.7%) diagnosed with a group stage of 0-2 and 35 patients (58.3%) diagnosed with a group stage of 3-4. The Pandemic and Vaccine-approved groups had 36 patients (28.1%) diagnosed with a group stage of 0-2 and 92 patients (71.9%) diagnosed with a group stage of 3-4; these results trended to statistically significant (P-value = 0.0688).

Conclusions

Our findings suggest that there have been a higher number of patients with head and neck cancer diagnosed with a T stage of 3 or 4 since the start of the COVID-19 pandemic. The effects of the COVID-19 pandemic are ongoing and will need further evaluation to determine the overall effects on oncology patients. Increased morbidity and mortality rates may be a potential result in the years to come.

## Introduction

The coronavirus disease 2019 (COVID-19) pandemic has affected all aspects of the healthcare system across the United States and abroad. In early 2020, increasing breakouts of the virus resulted in the reallocation of healthcare resources, stay-at-home orders, and fear of exposure at healthcare facilities among the public. Among the populations of high-risk patients, providers predicted a surge in oncology patients, likely with more advanced stage disease [1]. An advanced-stage disease can potentially result in more complex treatment options, more intensive and prolonged treatment, and a greater impact on the quality of life for the patients, making it imperative to start treatment as soon as possible after diagnosis.

Patients diagnosed with head and neck cancers should begin treatment especially quickly due to the chance of growth within the first three months [2], as well as the potential for airway compromise and the risk of severe bleeding [3]. Studies have shown a decrease in the number of head and neck cancer diagnoses and a delay in treatment initiation since the beginning of the pandemic [1,3-5]. With fewer consults and diagnoses throughout the pandemic, patients may present initially with more advanced-stage cancers and higher rates of more advanced-stage cancer might ultimately lead to higher morbidity and mortality rates for these patients. Multiple studies have suggested a higher rate of advanced-stage cancers [6-10], but overall impacts and survival rates have not yet been studied [3].

The impact of the COVID-19 outbreak appears to be evolving, and we may continue to see detrimental side effects in the months to come. In this study, we investigated how the COVID-19 pandemic has impacted the staging of head and neck cancers by comparing retrospective data from patients presenting in 2019, 2020, and 2021 at our institution. We hypothesized that there would be a higher incidence of more advanced-stage head and neck cancers due to the changes in cancer care during the pandemic.

## Materials and methods

A retrospective review of the medical records of 206 patients diagnosed with head and neck cancer at the University of Arkansas for Medical Sciences (UAMS) between August 19, 2019, and June 28, 2021, was performed. The patients were divided into three groups for analysis. The Pre-pandemic group included consults from 69 patients between August 19, 2019, and March 16, 2020, the Pandemic group included consults from 69 patients between March 17, 2020, and December 31, 2020, and the Vaccine-approved group included consults from 70 patients between January 1, 2021, and June 28, 2021. Collected data included date of consult, date of birth, race, hometown, disease site, tumor (T) stage, node (N) stage, M stage, group stage, receipt of surgery, and receipt of chemotherapy. American Joint Committee on Cancer (AJCC) eighth edition staging manual was used for T, N, and M staging. Disease sites include the sinus, oropharynx, oral cavity, nasopharynx, hypopharynx, larynx, salivary gland, thyroid skin, melanoma, and paraganglioma. The number of consults per month separated by year was compared. The three groups were compared according to T stage, N stage, M stage, group stage, and the type of treatment received. Group stages were classified as early stage (Stages 0, 1, and 2) versus advanced stage (Stages 3 and 4). There were 18 patients who had either a recurrence or a nonmalignant tumor. The types of treatment that were compared were patients that received radiation alone, radiation and surgery, radiation, surgery, and chemotherapy, and patients that declined radiation therapy.

## Results

A total of 206 patients were included in this retrospective chart review, which were then sorted into a Pre-pandemic group, a Pandemic group, and a Vaccine-approved group based on the date of the initial consult. The distribution of patients diagnosed with early-stage cancer and advanced-stage cancer for each group is demonstrated in Table 1, along with those diagnosed with a recurrence of previous cancer or a nonmalignant tumor.

**Table 1:** Patient and disease characteristics

Characteristic	Pre-Pandemic	Pandemic	Vaccine Approved
N	67	69	70
Early Stage			
0	2	0	0
I	14	10	12
II	9	8	7
TOTAL	25	18	19
Advanced Stage			
III	9	14	18
IV	26	29	30
TOTAL	35	43	48
Unknown Stage	7	8	3
Recurrence	6	7	2
Nonmalignant Tumor	1	1	1

**Figure 1:**
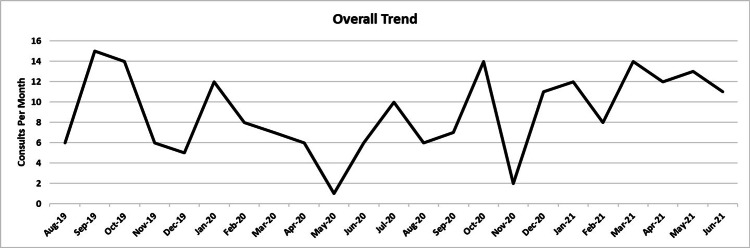
Comparing the total number of consults per month There was an overall decrease in the number of consults per month between January 2020 and May 2020. There were fewer consults per month during the year 2020 compared to the time periods between August 2019 and November 2019 and between January 2021 and June 2021. The graph shows the overall trend of consults per month between August 2019 and June 2021.

Figure 1 demonstrates the overall trend of the number of consults per month between August 2019 and June 2021. The largest decrease in the number of consults per month occurred between January 2020 and May 2020, decreasing from 12 consults per month to one consult per month. On average, the number of consults per month decreased from 10 consults per month during the Pre-pandemic period to seven consults per month during the Pandemic period and then increased to 12 consults per month during the Vaccine-approved period. Table 2 compares the consults per month separated by 2019, 2020, and 2021.

**Table 2:** Consults per month separated by 2019, 2020, and 2021

	2019	2020	2021
January		12	12
February		8	8
March		7	14
April		6	12
May		1	13
June		6	11
July		10	
August	6	6	
September	15	7	
October	14	14	
November	6	2	
December	5	11	

**Figure 2:**
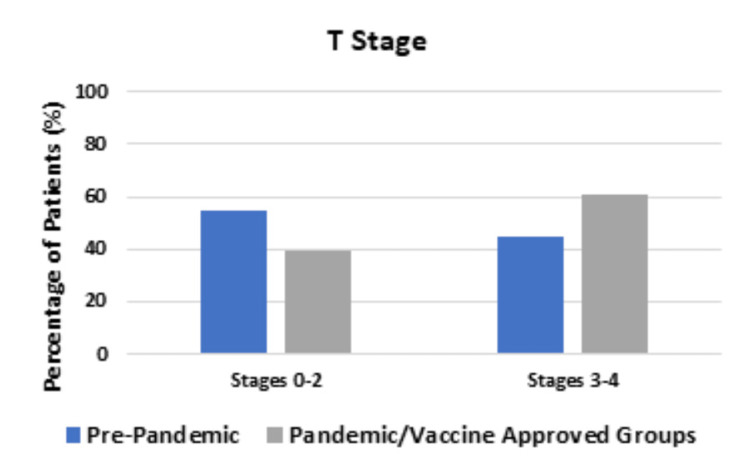
Comparing T stage between the Pre-pandemic group and the Pandemic and Vaccine-approved groups Comparing the groups, there was a higher percentage of patients in the Pre-pandemic group diagnosed with T0-T2 stage tumors compared to the Pandemic and Vaccine-approved groups diagnosed with T3-T4 stage tumors compared to the pre-pandemic group; this was statistically significant (P-value = 0.0426).

TNM staging distributions between the three groups were compared using Fisher’s exact tests. Figure 2 compares the T stage diagnosed for each of the three groups.

**Figure 3:**
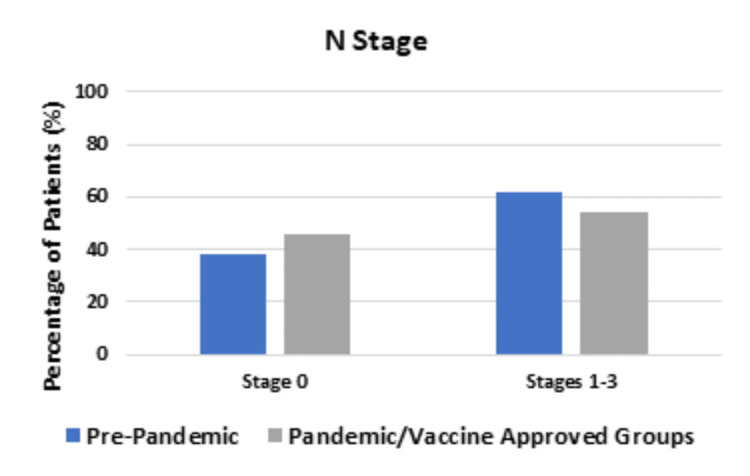
Comparing N stage between the Pre-pandemic group and the Pandemic and Vaccine-approved groups There was a higher percentage of patients diagnosed with N1-N3 stage tumors in the Pre-pandemic group compared to the Pandemic and Vaccine-approved groups, but this was not statistically significant (P-value = 0.3468).

**Figure 4:**
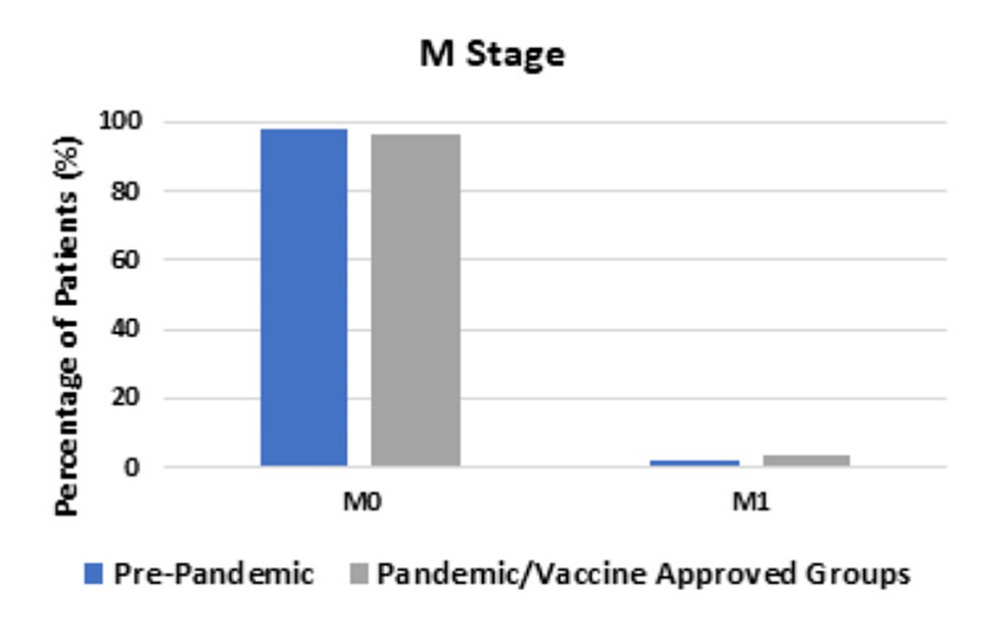
Comparing M stage between the Pre-pandemic group and Pandemic and Vaccine-approved groups In both groups, the majority of patients were diagnosed with M0 stage cancers. There was a slightly higher percentage of patients diagnosed with M1 stage cancer in the Pandemic and Vaccine-approved groups, but this was not statistically significant (P-value = 0.6662).

In the Pre-pandemic group, out of 67 patients, 33 (55.0%) were diagnosed with a T stage of 0-2 and 27 patients (45.0%) were diagnosed with a T stage of 3-4. In the Pandemic and Vaccine-approved groups, out of 139 patients, 50 patients (39.1%) were diagnosed with a T stage of 0-2 and 78 patients (60.9%) were diagnosed with a T stage of 3-4; these differences were statistically significant (P-value = 0.0426). There was no statistical difference between the groups in the N stage or the M stage, as shown in Figure 3 and Figure 4, respectively.

**Figure 5:**
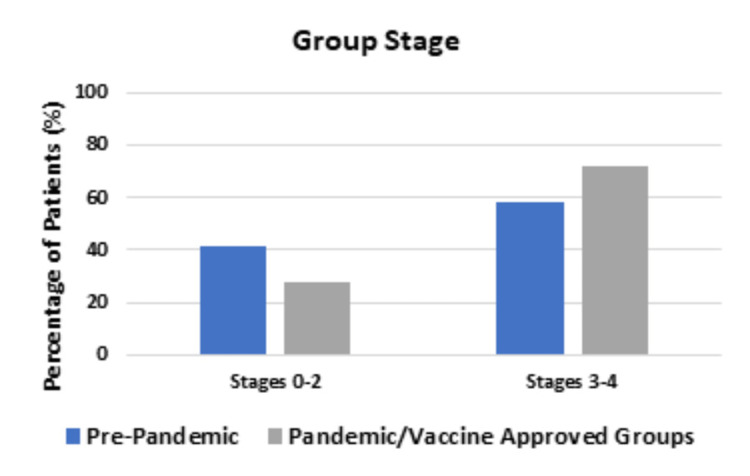
Comparing the group stage between the Pre-pandemic group and Pandemic and Vaccine-approved groups A higher percentage of patients presented with Group 3 and 4 stage cancers in the Pandemic and Vaccine-approved groups compared to the Pre-pandemic group; this result trended to statistical significance (P-value = 0.0688).

Figure 5 depicts the differences in the group stage diagnosed in the Pre-pandemic group and the Pandemic and Vaccine-approved groups.

The Pre-pandemic group had 25 patients (41.7%) diagnosed with a group stage of 0-2 and 35 patients (58.3%) diagnosed with a group stage of 3-4. The Pandemic and Vaccine-approved groups had 36 patients (28.1%) diagnosed with a group stage of 0-2 and 92 patients (71.9%) diagnosed with a group stage of 3-4; these results trended to statistical significance (P-value = 0.0688).

## Discussion

The COVID-19 pandemic has impacted cancer care, diagnosis, and treatment in ways that are still not fully understood, but medical providers have already and will continue to see the lasting effects on their patients for years to come. The number of diagnostic procedures for detecting cancer and patients diagnosed with several different types of cancer, including breast, colorectal, lung, pancreatic, and gastric cancers, was lower than the numbers prior to the pandemic [11]. Specifically for head and neck cancers, two studies demonstrated a decrease in the number of patients presenting for care of head and neck cancers in the early months of the pandemic [9,12]. Our study showed similar findings; there was a decrease in the number of consults per month between January 2020 and May 2020, decreasing from 12 consults per month to 1 consult per month.

In addition, the results of our study confirm our original prediction that there would be a higher incidence of patients presenting with more advanced stage head and neck cancers since the pandemic started compared to before the pandemic. More advanced-stage cancers are associated with worse outcomes and more complicated treatment plans for patients [1]. A study investigating the delayed presentation of head and neck cancer patients during the pandemic concluded there was an increased number of admissions with advanced disease and an increased need for more complex surgical procedures likely due to a delay in seeking out care and reallocation of resources [3]. There is evidence that shows patients with advanced stage head and neck tumors were more likely to report cancer-related complications such as pain, edema, and self-medication [5]. Based on practice recommendations published by the American Society of Radiation Oncology and the European Society for Radiotherapy and Oncology, head and neck oncologists had to balance increased requirements for nonsurgical treatments due to operating room closures, altered risk-benefit ratio of chemotherapy and radiation therapy as a result of increased susceptibility for severe acute respiratory syndrome coronavirus 2 (SARS-CoV-2) infection, a need to suppress the spread of coronavirus, and a shortage of radiation therapy resources [13].

Based on the results from Fisher’s exact tests, this study does not show a statistically significant difference between the group stage of patients with head and neck cancer before and after the beginning of the pandemic. This result could be due to the small sample size or insufficient time to evaluate. The long-term effects of the pandemic are still unclear and will likely take many years to determine the overall effect on different patient populations. There is a statistically significant difference in the T stage diagnosed before and after the pandemic, which suggests we are only seeing the beginning of the true outcomes that this pandemic has had on cancer patients. Based on the trends, there was a sharp drop in the number of patients seen for consultation in the early months of the pandemic (March-May 2020) compared to the previous year (Figure 1). This implies patients were less likely to be seen in the clinic and diagnosed early, giving a possible explanation for why there were a higher number of patients presenting with more advanced-stage cancers now.

Our results are mostly consistent with the initial experiences published at the beginning of the pandemic [1,3-5]. For example, a study of patients from Turkey determined there was a higher rate of T3-4 tumors and an increased number of patients presenting with more locoregionally advanced disease during the early months of the pandemic (March-September 2020) compared to the same period from 2019, but overall impacts on survival rates had not yet been studied [3]. Similar findings were seen in patients presenting at a major cancer center in the United States; there was a statistically significant increase in tumor size and T stage seen in the early months of the pandemic (May-June 2020) compared to the same period from 2019 [12]. In addition, in a study specific to oral cancers, an analysis of patients at a tertiary care hospital in South India found a statistically significant increase in advanced T stage from April 2020 through June 2020 compared to the same period from 2019 [9]. The same was also true in a series of oral cavity cancer patients treated at Heidelberg University Hospital in Germany in 2020 when compared to patients from 2010-2019 [8]. However, another study from the University Hospital Erlangen in Germany of patients diagnosed from April 2020 to April 2021 did not find a difference in the number or severity of head and neck squamous cancers when compared to March 2019 to March 2020 [14].

Managing the diagnosis and treatment of cancer patients changed in many ways at the beginning of the pandemic, but those patients diagnosed with cancer who then tested positive for SARS-CoV-2 faced additional barriers. Several studies have shown that cancer patients are more vulnerable to having severe illness and high mortality rates from COVID-19 [15]. One prospective cohort study analyzed specific factors associated with cancer treatment delay among patients diagnosed with COVID-19 and found that individual patient factors, social determinants of health, and COVID-19 severity and diagnosis date were associated with exacerbated health disparities in regard to cancer treatment delay [16]. The same study also noted that individuals who contracted COVID-19 later in the pandemic were associated with a lower likelihood of treatment delay and reduction in delays when compared with individuals who contracted COVID-19 during the months of March to June 2020, indicating better patient outcomes with increased understanding of the virus [16]. Oncologists were also faced with the decision of how to treat COVID-19-positive patients versus COVID-19-negative patients. In one European survey, most respondents recommended standard radiotherapy treatment for COVID-19-negative patients and favored a delay in radiotherapy treatment or shorter fractionation for COVID-19-positive patients [17].

Delays in cancer care and diagnosis can drastically alter the outcome for patients, which is why continuity of care and access to care are crucial [1]. A study investigating the delays and disruptions in cancer care concluded that most were caused by a reduction in routine activities of cancer services, a reduction in cancer surgeries, delays in radiation therapy, and the rescheduling or cancellation of outpatient visits [18]. While there were obvious short-term disruptions in the care provided to cancer patients during the pandemic, there have also been unintended consequences that have the potential to cause long-term morbidity and survival implications for patients who have gone without care during this period [19]. There will have to be an effort by the entire healthcare system to change the trajectory for both newly diagnosed and long-term cancer patients in the following years.

Multiple lessons should be kept in mind for a roadmap should another pandemic occur in the next years/decades. First, maintaining access to care will be very important. While clinics and operating rooms were shut down at the pandemic's beginning, cancer patients experienced delays in diagnosis, workup, and definitive treatment [20]. The rapid development of a diagnostic test for the infectious agent for the next pandemic will be critical to minimize closings in clinics and hospitals. While clinics and hospitals may be closed in the early days of the next pandemic, telemedicine services should be utilized to allow patients to be assessed expediently [21]. Fortunately, there has been a dramatic increase in telemedicine service availability and utilization [22]. Finally, ongoing health education will be essential to inform the public of the importance of cancer screening, workup, diagnosis, and treatment, even during a pandemic.

There are several limitations to our study. First, the retrospective nature of the study must be acknowledged. Second, the population of patients seen at our academic institution may not represent or be generalizable to other practice types or locations. This is especially true given the variability in COVID-19 incidence rates in different locations, as well as differences in resources and responses from local, regional, and national governments. These limitations highlight the need for multicenter, national, and global analyses to define the extent and scope of the problem.

## Conclusions

Our study highlights that there is a correlation between advanced stage head and neck cancers and the COVID-19 pandemic. The results do not account for any delay in treatment and do not specifically investigate the reasons why some patients might have chosen to postpone coming to the hospital sooner. It is possible that we are only seeing the beginning of the overall clinical effects of the pandemic on cancer patients. It is crucial for these patients to be recognized so that proper mitigation strategies can be implemented to reduce these effects in the years to come and, ultimately, decrease the chance of higher morbidity and mortality rates in cancer patients.
